# Nucleotide Weight Matrices Reveal Ubiquitous Mutational Footprints of AID/APOBEC Deaminases in Human Cancer Genomes

**DOI:** 10.3390/cancers11020211

**Published:** 2019-02-12

**Authors:** Igor B. Rogozin, Abiel Roche-Lima, Artem G. Lada, Frida Belinky, Ivan A. Sidorenko, Galina V. Glazko, Vladimir N. Babenko, David N. Cooper, Youri I. Pavlov

**Affiliations:** 1 National Center for Biotechnology Information, National Library of Medicine, National Institutes of Health, Bethesda, MD 20894-6075, USA; frida.belinky@gmail.com; 2 Center for Collaborative Research in Health Disparities–RCMI Program, Medical Sciences Campus, University of Puerto Rico, San Juan, PR 00936, USA; abiel.roche@upr.edu; 3 Department Microbiology and Molecular Genetics, University of California, Davis, CA 95616, USA; alada@ucdavis.edu; 4 Institute of Cytology and Genetics, Novosibirsk 630090, Russia; vanyasidorenko22@gmail.com (I.A.S.); babenko@yahoo.com (V.N.B.); 5 Department of Biomedical Informatics, University of Arkansas for Medical Sciences, Little Rock, AR 72205, USA; GVGlazko@uams.edu; 6 Institute of Medical Genetics, Cardiff University, Cardiff CF14 4AY, UK; CooperDN@cardiff.ac.uk; 7 Departments of Microbiology and Pathology; Biochemistry and Molecular Biology; Genetics, Cell Biology and Anatomy, University of Nebraska Medical Center, Omaha, NE 68198, USA; 8 Eppley Institute for Research in Cancer and Allied Diseases, Omaha, NE 68198, USA

**Keywords:** DNA sequence profile, Monte Carlo, mixture of normal distributions, somatic mutation, tumor, mutable motif, activation induced deaminase, AID/APOBEC

## Abstract

Cancer genomes accumulate nucleotide sequence variations that number in the tens of thousands per genome. A prominent fraction of these mutations is thought to arise as a consequence of the off-target activity of DNA/RNA editing cytosine deaminases. These enzymes, collectively called activation induced deaminase (AID)/APOBECs, deaminate cytosines located within defined DNA sequence contexts. The resulting changes of the original C:G pair in these contexts (mutational signatures) provide indirect evidence for the participation of specific cytosine deaminases in a given cancer type. The conventional method used for the analysis of mutable motifs is the consensus approach. Here, for the first time, we have adopted the frequently used weight matrix (sequence profile) approach for the analysis of mutagenesis and provide evidence for this method being a more precise descriptor of mutations than the sequence consensus approach. We confirm that while mutational footprints of APOBEC1, APOBEC3A, APOBEC3B, and APOBEC3G are prominent in many cancers, mutable motifs characteristic of the action of the humoral immune response somatic hypermutation enzyme, AID, are the most widespread feature of somatic mutation spectra attributable to deaminases in cancer genomes. Overall, the weight matrix approach reveals that somatic mutations are significantly associated with at least one AID/APOBEC mutable motif in all studied cancers.

## 1. Introduction

The sequencing of genomes of solid tumors and liquid malignancies associated with different types and stages of cancer has revealed a plethora of genetic changes, from nucleotide substitutions and insertions/deletions to chromosomal rearrangements and chromosome copy number alterations [1,2,3]. As predicted decades ago by the mutator theory of cancer [4], the elevated mutability in tumors contributes both to their onset and to their further evolution. The underlying causes of this mutagenesis are diverse, from the appearance of mutator mutations to DNA damage by intrinsic or environmental mutagens (e.g., oxidative stress, tobacco smoke, UV light, etc.) [5]. Somatic genome instability leads to the activation of oncogenes and inactivation of tumor suppressors and helps tumor cells to emerge, proliferate, elude immune surveillance, and acquire resistance to anticancer drugs.

In some cancers, the number of single nucleotide variations (SNVs) is in the order of tens of thousands per genome. A few driver mutations [6,7] ultimately lead to cancer, while the role, if any, of the vast majority of mutations, termed “passengers”, during tumor development is poorly understood [8,9]. One crucial principle stands out: mutations can be classified into ‘families’ based upon their flanking DNA sequences [10,11]. Different mutagenic processes generate mutations within different contexts of a neighboring nucleotide sequence (the bases upstream and/or downstream of the mutations, termed “mutation signatures”). Sophisticated approaches have been developed to extract the most prominent signatures from a complex mix of mutational targets resulting from the action of a variety of mutagens, both exogenous and endogenous, operating during tumor evolution [12,13]. Both driver and passenger mutations have been used in the analysis. One of the clearest mutational signatures, found in breast and other cancers [14,15], is characterized by C:G to T:A or C:G to G:C substitutions that are found predominantly in the 5′-TC sequence motif (signatures #2 and 13; listed in the COSMIC database). These signatures have been attributed to the action of nucleic acid-editing enzymes, cytosine deaminases. These enzymes, collectively called APOBECs, deaminate cytosine in single-stranded DNA, yielding uracil. DNA replication past the uracil leads to the insertion of A, thereby giving rise to the C-to-T transition. Also, abasic sites that are produced as intermediates of uracil repair are bypassed by the cytidine transferase activity of REV1 translesion DNA polymerase, leading to C:G to G:C transversions. Cytosine deaminases possess inherent sequence specificity. Thus, for example, activation induced deaminase (AID) prefers to deaminate within 5′-WRC motifs (W = A or T, R = A or G), whereas APOBEC3G acts preferentially on the last cytosine in the 5′-CCC motif, while two other APOBEC3 enzymes, APOBEC3A and APOBEC3B, exhibit a preference for 5′-TC sequences. Another prominent feature of APOBEC enzymes is their ability to act in a processive fashion, i.e., to catalyze multiple deamination events per substrate-binding event [16], thereby inducing kataegis (clustered mutations); however, it should be noted that APOBEC action is only one possible explanation for kataegis in cancer cells [17]. Mutational signatures of cytosine deaminases are detected in many cancers [15]. It is unlikely to be a mere coincidence that the APOBEC3 enzymes are frequently upregulated in tumors [18,19]. It should be noted that if deaminases act on 5-methylcytosine generating “T”, a specialized G:T mismatch repair mechanism operates, and the genetic consequences could be different because of the disappearance of an epigenetic mark [20]. There is evidence for the contribution of this process to cancer [21]. 

Cancer genome studies necessitate working with huge datasets; the obvious problems posed by the analysis of such data are partially solved by the advent of the “mutational signature” technique [12,22,23]. It is not usually possible to define the DNA strand upon which the vast majority of mutations has occurred (but see [24,25]); for example, both a C>T change on one strand and a G>A change on the opposite strand lead to the same CG to TA transition. Therefore, in practice, the analysis may be reduced to the study of only six different types of substitution. Similarly, there are 96 context-dependent mutations (mutation types) that consider two nucleotides in the flanking 5′ and 3′ positions of the mutated nucleotide [23]. Analysis of the mutational spectra of context-dependent mutations in cancer genomes involves pooling all the mutations from cancer samples into a discrete distribution according to the mutation types, while further analysis involves the so-called non-negative matrix factorization (NMF) method [12,22,23]. There are some variations of this basic technique; indeed, Temiz et al. [26] presented a 32 × 12 mutation matrix, which captures the nucleotide pattern two nucleotides upstream and downstream of each mutation. In this study, a somatic autosomal mutation matrix (SAMM) representing tumor-specific mutations and mechanistic template mutation matrices (MTMMs) representing oxidative DNA damage, ultraviolet-induced DNA damage, (5m)CpG deamination, and APOBEC-mediated cytosine mutation were constructed. MTMMs were mapped to the individual tumor SAMMs to identify mutational mechanisms corresponding to each overall mutational pattern. The method appeared to be sensitive enough to retrospectively allocate the origins of tumors to specific tissues [26].

In an attempt to increase the specificity and sensitivity of the arsenal of techniques available for mutation analysis in whole genomes, we have employed mutable motifs of cytosine deaminases represented in the form of weight matrices (sequence profiles) [27,28,29]. This approach may be expected to be a more general descriptor of nucleotide sequences as compared to the sequence consensus approach, because it takes into account the variability in the information content (“conservation”) across neighboring positions. Control experiments using various constrained samples of randomly selected sequences indicated that the level of false positives obtained using this approach is even lower than the expected false discovery rate (~0.05, see Section 4.5, Section 4.6, Section 4.7 and Section 4.8 for details). These analyses suggest that the weight matrices method is a powerful tool for the analysis of genomic mutations. Further, we identified prominent mutational footprints of APOBECA and APOBECB in many human cancers. Mutable motifs attributable to AID are less pronounced but are nevertheless present ubiquitously in cancer genomes. 

## 2. Results

### 2.1. Weight Matrices of AID/APOBEC Mutable Motifs

**Figure 1:**
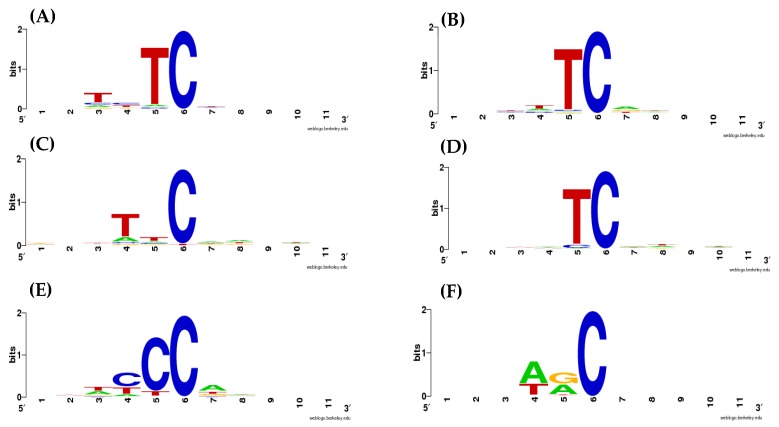
Information content and derived consensus sequences of the DNA context of mutations induced by AID/APOBEC deaminases in yeast genomes (frequencies of nucleotides were used as input). (**A**) APOBEC1, (**B**) APOBEC3A, (**C**) APOBEC3C, (**D**) APOBEC3B, (**E**) APOBEC3G, and (**F**) AID. Position 6 is the position of the somatic mutations. AID/APOBEC weight matrices are shown in Supplementary Figure S1.

The information content of AID/APOBEC mutable motifs is shown in Figure 1 (the list and sources of the mutated sequences are shown in Supplementary Table S1). AID/APOBEC cytosine deaminases exhibit substantial variability in terms of their mutable motifs. T in position −1 (number 5 in Figure 1) was the most prominent feature of the APOBEC1, APOBEC3A, and APOBEC3B enzymes, consistent with previous studies (reviewed in [23]). APOBEC3C has a distinct mutable motif with T in position −2. Additionally, APOBEC1 has an excess of T in position −3 (number 3 in Figure 1).

APOBEC3G has a distinct mutation pattern wcCCw (lower case w and c mean substantially lower information content as compared with the upper case, Figure 1), which is a variation of the previously described CCC motif and CCR motif [7,30]. The AID deaminase has the expected context specificity, WRC [16,31]. 

It is hard to demarcate the mutational signatures of APOBECs using the consensus approach due to the high variability of information content across sites. For example, APOBEC3G has a highly conserved C in positions −4 and −5; however, there is also a less conserved C (and lower information content) in position −3 that may or may not be included in a consensus sequence (Figure 1). We opted to employ the widely used weight matrix technique (see Section 4) in order to avoid uncertainties with the less informative positions. 

We compared the nucleotide composition of mutation sites (±5 nucleotides, Supplemental Figure S1) for all the studied AID/APOBEC proteins using the χ^2^ test (Table 1). We found that all six AID/APOBEC proteins studied were significantly different with respect to the DNA sequence context of the mutation sites expressed in the form of nucleotide frequency matrices (Table 1). Thus, weight matrices properly represent the DNA sequence context of mutations induced by various AID/APOBEC proteins, as noted in previous studies [5] where a simple consensus approach was used. We aimed to differentiate between the mutable motifs associated with the various AID/APOBEC proteins, although this was not always possible (for example, the sequence contexts of the APOBEC3A, APOBEC3B, and APOBEC3C targets are not as different as other pairwise comparisons, see Table 1).

We performed four control experiments (for details, see Section 4.5, Section 4.6, Section 4.7 and Section 4.8): (1) analysis of the sequence context of somatic mutations in mitochondrial DNA as a negative control [32]; (2) analysis of the correlation between the matrices of shuffled sites of mutations and the sites of somatic mutation in cancer cells using the expected false discovery rate approach [33]; (3) analysis of the correlation between matrices of randomly sampled sites from the yeast genome and somatic mutations in cancer cells using the expected false discovery rate approach [33]; and (4) analysis of somatic mutations in human immunoglobulin genes as a positive control [34,35,36]. The results of all four control experiments (Supplementary Tables S2–S5) strongly support our contention that the weight matrix technique is applicable to the studied AID/APOBECs (for details, see Section 4).

### 2.2. Analysis of the Correlation between AID/APOBEC Mutable Motifs and Somatic Mutations in Cancer Cells: C:G>T:A Transitions

**Figure 2:**
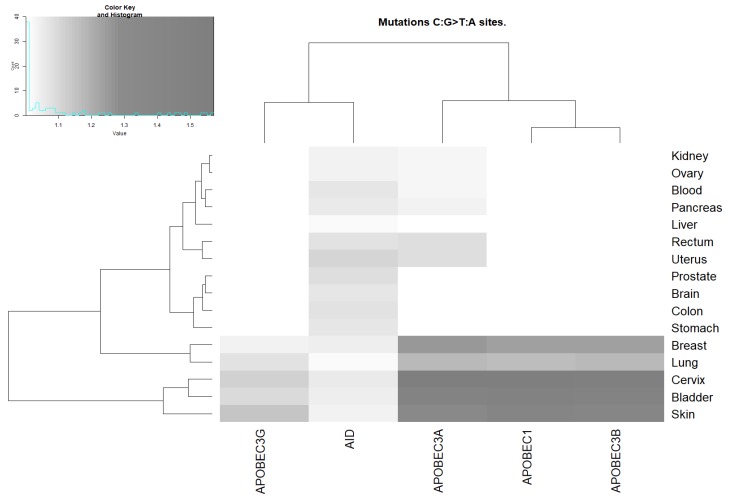
Correlation between AID/APOBEC mutable motifs and the sequence context of somatic C:G>T:A mutations. For the actual data, see Supplementary Table S6. The intensities of the gray color correspond to the ratio values (the ratio being the mean weight of the mutated sites divided by the mean weight of the non-mutated sites). The unweighted pair group method with arithmetic mean (UPGMA) clustering of ratio values for the AID/APOBEC footprints and tissues is shown as dendrograms.

We examined the correlation of the sites of C:G>T:A mutations in cancers and AID/APOBEC mutable motifs. A correlation between a mutable motif and the DNA context of somatic mutations from the COSMIC database was claimed when the results of two statistical tests (Monte Carlo test and *t*-test, see Section 4) were both significant. A correlation between the mutable motifs of (at least one) deaminase(s) and the sites of somatic C:G>T:A mutations was found for all cancer tissues (Figure 2 and Supplementary Table S6). AID activity was the most ubiquitous according to the enzyme characteristic signature in various cancer types, whereas the APOBEC1, APOBEC3A, APOBEC3B, and APOBEC3G signatures were detected less frequently, although their signatures were stronger, most notably in breast, lung, cervix, skin, and bladder cancer (Figure 2).

We attempted to estimate the fraction of somatic mutations associated with AID/APOBEC deamination using a mixture of two normal distributions (see Section 3 and Section 4 for details). For example, estimated fractions of APOBEC1-associated mutations (0.66, 0.48, 0.74, 0.39, and 0.62) look consistent with the smallest value of 0.39 corresponding to the lowest ratio value (1.064, APOBEC1, lung), although this method sometimes yielded potentially underestimated values (0.17, APOBEC3G, cervix, ratio = 1.113) and overestimated values (0.92, APOBEC3G, bladder, ratio = 1.101) (Supplementary Table S6). The overall distribution of fractions for APOBEC1, APOBEC3A, ABOPECB, and AID deaminases is shown in Supplementary Figure S2. The mean of the fractions in Figure S2 is 0.42 (Supplementary Table S6). This result suggests that a substantial proportion of somatic mutations is associated with AID/APOBEC mutagenesis.

### 2.3. Analysis of the Correlation between AID/APOBEC Mutable Motifs and Somatic Mutations in Cancer Cells: C:G>G:C and C:G>A:T Transversions

**Figure 3:**
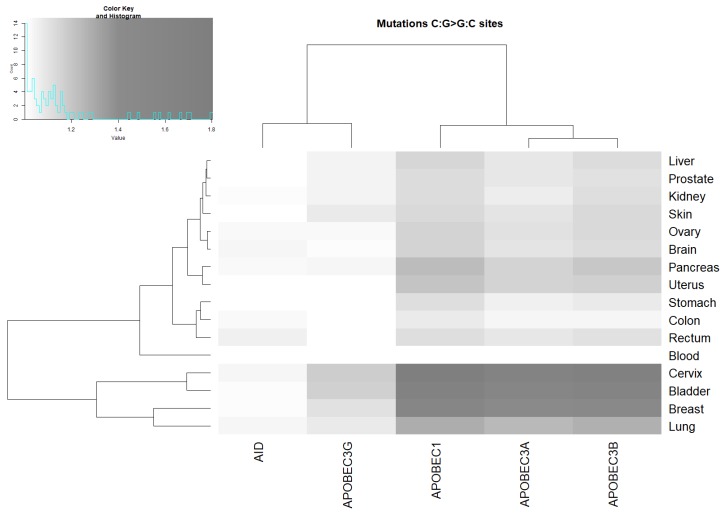
Correlation between AID/APOBEC mutable motifs and the sequence context of somatic C:G>G:C mutations. For actual data, see Supplementary Table S7. The intensities of the gray color correspond to the ratio values (the ratio being the mean weight of the mutated sites divided by the mean weight of the non-mutated sites).

Many C:G > G:C transversions were suggested to be the result of processing abasic sites after the removal of uracils originating via DNA deamination by AID/APOBEC proteins [37]. Consistent with this idea, a significant correlation of these mutations with mutable motifs was found in many cancers (Figure 3 and Supplementary Table S7). The transversions associated with APOBEC1, APOBEC3A, and APOBEC3B were found to be more abundant in comparison with APOBEC3G and AID, suggesting a role of these three deaminases in generating C:G>G:C somatic mutations in human cancer. The correlation with the three APOBEC motifs was again strongest for breast, bladder, cervix, and lung cancer.

**Figure 4:**
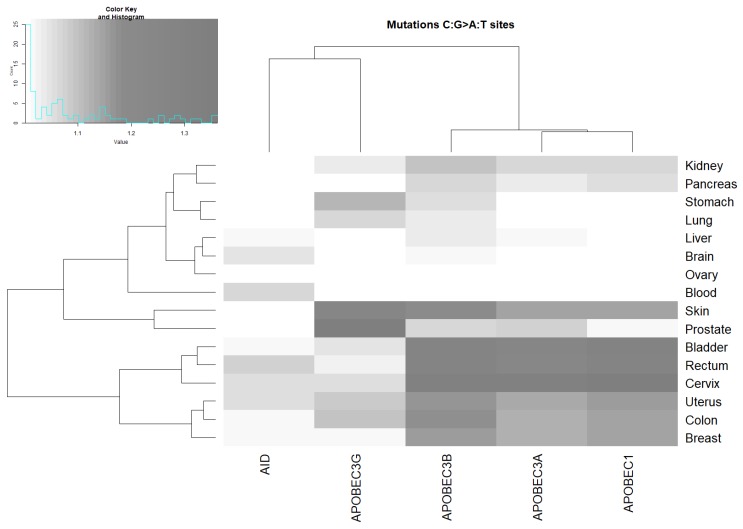
Correlation between AID/APOBEC mutable motifs and the sequence context of somatic C:G>A:T mutations. For actual data, see Supplementary Table S8. The intensities of the gray color correspond to the ratio values (the ratio being the mean weight of the mutated sites divided by the mean weight of the non-mutated sites).

Although it has been proposed that C:G>A:T mutations are a less likely outcome of AID/APOBEC enzymatic action, we found a significant excess of these transversions in many cancers (Figure 4 and Supplementary Table S8), suggesting that a significant portion of C:G>A:T mutations may be caused by processes initiated by deamination by AID/APOBEC enzymes. That the APOBEC3A, APOBEC3B, and APOBEC3G footprints are more abundant in comparison with the APOBEC1 and AID motifs suggests an important role for these three deaminases in generating somatic C:G>A:T mutations in human cancers.

The unweighted pair group method with arithmetic mean (UPGMA) clustering of ratio values for AID/APOBEC footprints and tissues (Figure 2, Figure 3 and Figure 4) suggests that AID/APOBEC3G form one clade, whereas APOBEC1/3A/3B form another clade according to the distributions of the ratios across tissues (graphs above heatmaps at Figure 2, Figure 3 and Figure 4). This can be explained by the high similarity of APOBEC1/3A/3B signatures (Figure 1). Breast, bladder, and colon tend to form a separate group according to the distributions of ratios across the AID/APOBEC footprints (graphs above heatmaps at Figure 2, Figure 3 and Figure 4). In general, these classifications are not consistent, reflecting large variations in transition/transversion ratios (Supplementary Tables S6–S8) and are likely to be a result of variation in the efficiency of DNA repair of such sites in different tissues [5,36,38].

### 2.4. Analysis of Various Tumor Types in Blood and Skin

Cancers of the blood system were found to be associated with AID and APOBEC3A (Figure 2, Figure 3 and Figure 4 and Supplementary Tables S6–S8). No other putative associations with APOBEC enzymes were identified. We performed an analysis of two blood cancer subtypes with the highest representation in the COSMIC dataset (see Section 4): acute myeloid leukemia and germinal center B-cell-like (GCB) lymphomas (Table 2). A significant excess of somatic mutations in AID mutable motifs was detected in acute myeloid leukemia (Table 2). In GCB lymphomas, a significant excess of somatic mutations was detected in both AID and APOBEC3A mutable motifs (Table 2). These results suggest that there is variability of mutation context specificity across the same tissue, as seen previously [39].

We also performed an analysis of two skin cancer subtypes with the highest representation in the COSMIC dataset (see Section 4.3) (Table 2): skin cutaneous melanoma and skin adenocarcinoma. Both tumor types yielded somewhat similar results. An overwhelming excess of somatic mutations in APOBEC1 and APOBEC3A/B/G mutable motifs (Table 2) is likely to be due to the known excess of mutations in dipyrimidine dinucleotides (for example, TC) in skin cutaneous melanoma caused by mutagenic UV photoproducts [40]. Accordingly, we interpreted the excess of mutations in the AID/APOBEC3A/B/G contexts (Table 2) to be the result of false positives (as was already suggested by the results of the control experiments; for details, see Section 4.7), but we are also aware of evidence for the direct role of deaminases in skin cancer [41]. We observed a much lower excess of mutations in the mutable motifs observed in skin adenocarcinoma (Table 2). These results are likely to reflect the participation of AID/APOBEC deaminases in mutagenesis, because UV photoproducts do not play any role in the mutagenesis of skin adenocarcinomas [39]. Thus, APOBECs may play a role in a proportion of cases of squamous cell carcinoma [42].

**Figure 5:**
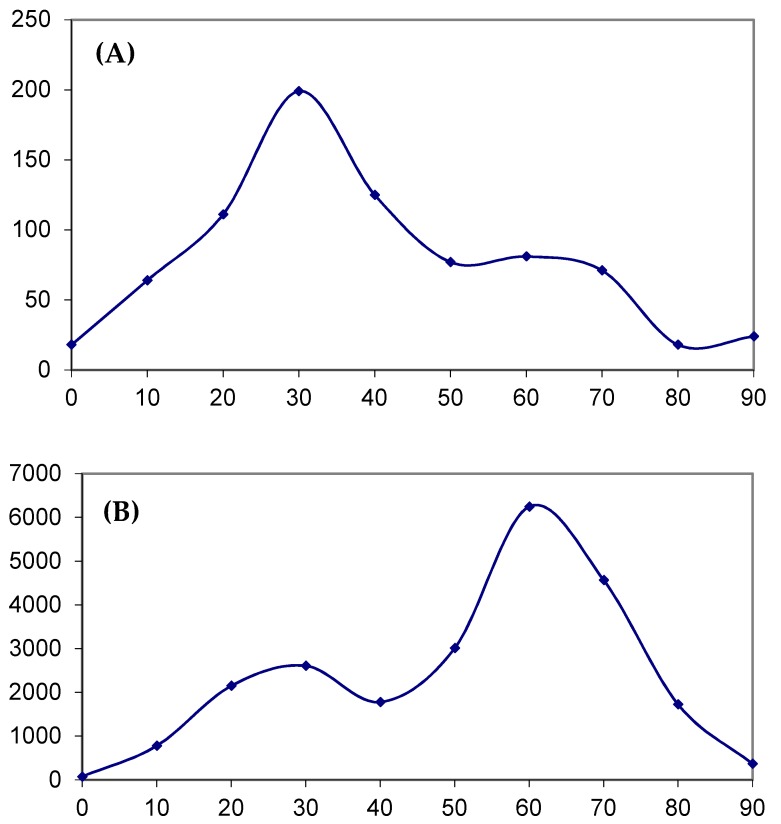
The weight distribution obtained using (**A**) the APOBEC3G weight matrix for skin adenocarcinoma (Table 2) and (**B**) the APOBEC1 weight matrix for bladder tissue (Supplementary Table S6). X axis: 0 stands for 0–9 interval of weights, 1 stands for the 10–19 interval, 2 stands for 20–29, etc.

The mixture of two normal distributions yielded fairly predictable results (0.168–0.687, Table 2, see Section 2.2) except for the APOBEC3G mutable motifs in skin cutaneous melanoma samples where the fraction of sites potentially associated with the APOBEC3G mutable motifs is extremely large (0.982, Table 2). The distribution of weights for this case is shown in Figure 5A. A putative component (normal distribution) corresponding to the APOBEC3G mutable motifs (large weights, the rightmost distribution) was less obvious compared with Figure 5B, which can be classified as a reasonable result, because the fraction of sites potentially associated with the APOBEC1 mutable motifs (0.65) is close to the mean of the fractions estimated above (0.42, Supplementary Figure S2). This distorted normal distribution (another problem is a much larger number of sites in the last bin compared with the previous bin) may be a reason why two distributions (Figure 5A) were incorrectly classified (mixed together) yielding an obvious overestimate for the APOBEC3G mutable motifs (see Section 3). This is a known problem in classification analyses of this kind [43,44].

## 3. Discussion

The advantage of our approach is that we used a unified computational technique that allowed an objective and accurate comparison of the mutational contribution of various APOBEC enzymes under the same experimental conditions and for the same datasets. We confirm that while the mutational footprints of APOBEC1, APOBEC3A, APOBEC3B, and APOBEC3G are prominent in many cancers, mutable motifs characteristic of the humoral immune response somatic hypermutation machine, AID, are the most widespread feature of the somatic mutation spectra attributed to APOBECs in cancer genomes. It is important to note that the suggested technique does not depend on expert opinion as to the exact consensus sequences and, therefore, objectively represents mutable motifs. 

Somatic mutations in all 18 studied cancer types are significantly associated with at least one AID/APOBEC mutable motif. The blood subset of mutations stands apart because only AID mutable motifs are detected (Figure 2, Figure 3 and Figure 4 and Table 2). Although there are significant differences between the contexts of AID/APOBEC-induced mutations manifested in frequency matrices (Table 1), there are many tissues where mutable sites have been found to be targeted by two or more deaminases (Figure 2, Figure 3 and Figure 4). In such cases, we cannot reliably differentiate between different deaminases with similar mutable motifs (Figure 1). For example, the frequency matrices of APOBEC1, APOBEC3A, and APOBEC3B are quite similar to each other (Figure 1 and Table 1), and this represents a major problem. To resolve this issue, it may be possible to use additional information, for example, gene expression data. However, the addition of expression data was not particularly informative for the AID and DNA polymerase η mutational footprints [21,39]. The same conclusion was reached in several other studies, because the genomic level of cytosine deamination does not necessarily correlate with the expression of the corresponding AID/APOBEC genes [15,23,45]. For this reason, we did not attempt to compare expression data from different tissue types and relate these data to the results we obtained.

In order to take into account the differences in the base composition between the yeast and human genomes, we used the simplest normalization procedure by taking the frequencies of nucleotides in the non-informative positions −5, −4, +4, and +5 as a null model (Figure 1, see Section 4.7 for details). Although the control experiments suggest that this normalization tends to yield results that are consistent with our expectations (with the exception of bladder, cervix, and skin tumors; see Section 4.7), we cannot exclude the possibility that more sophisticated normalization schemes might be required to generate more accurate results.

In addition, the role of APOBEC3C in mutagenesis remains uncertain and requires further investigation. Another potential methodological problem (at least, for complex computational techniques) is that we have a “positive” set (sites of mutations: sites that contain characteristic features of mutable motifs) and do not have a “negative” set (sites of mutations: sites that do not contain characteristic features of mutable motifs). Randomly sampled sites from yeast chromosomes are far from being a good “negative” set, because distributions of mutations across yeast chromosomes are too sparse and may contain a lot of mutable motifs. This is not a problem for the weight matrix technique, which does not use negative sets as a part of its learning procedures. However, this is the major problem for more sophisticated methods. For example, it is an obstacle for the application of supervised learning methods (e.g., hidden Markov models or support vector machine), because the training of these artificial intelligence (AI) algorithms requires classified or labeled data. However, unsupervised learning methods (such as k-means clustering), which do not need classified data, may be applied to this problem. Another issue is the need to take into account the much higher A:T content of the mutation sites in the yeast genome as compared with the human genome; this should be implemented as a part of a learning procedure.

The results of all the control experiments and somatic mutations in cancers strongly suggest that the weight matrix technique is applicable to various types of mutational signatures. The suggested approach complimented with clustering techniques (Figure 2, Figure 3 and Figure 4) allows for comparison between the studied enzymes and tissues. The suggested approach can be applied to various exciting questions in cancer genomics, including the underlying causes of the non-uniform distribution of somatic mutations across the human genome and asymmetries of mutagenesis with respect to leading/lagging and non-transcribed/transcribed DNA strands.

We estimated the impact of mutagenesis associated with AID/APOBEC deamination by representing distributions of weights as mixtures of two normal distributions. This approach is based on the method of estimating the protein coding density in a corpus of DNA sequence data, in which a ‘protein-coding coding statistic’ (which is similar to distributions of weights of somatic mutation contexts) is calculated for a large number of windows for the sequences under study, and the distribution of the statistic is decomposed into two normal distributions, assumed to be the distributions of the coding statistic in the coding and non-coding fractions of the sequence windows [43]. The distribution with the largest mean was assumed to reflect the fraction of protein coding fragments [43]. Similarly, the fraction of sites in a distribution with the largest mean was assumed to be the fraction of mutations induced by the AID/APOBEC enzymes. We noted problems with such an approach for some cases (see Section 2.4). However, the method tends to produce reasonable estimates. Rare deviations from normality caused by the natural boundaries of weight distributions (0 and 100, see the last bin in Figure 5A) is a possible explanation for the problems associated with the use of this classification technique in some cases.

Our analysis suggested that initial deamination events lead to both transitions and transversions. This is already known for somatic mutations initiated by AID in immunoglobulin genes and for APOBEC enzymes in cancer [5,38]. A large variation in transition/transversion ratios (Supplementary Tables S6–S8) is likely to be a result of peculiarities in the relative abundance of proper DNA substrates for deamination and the various efficiency of the DNA repair of such sites [5,36,38]. Overall, our results suggest that AID/APOBEC proteins make a major contribution to several different types of somatic mutations in cancer. The idea that APOBECs can be carcinogenic was originally proposed by Neuberger et al. in early 2000s [46], after the discovery that these proteins can edit DNA [47,48] and, therefore, are by definition mutators. Under normal conditions, deaminases are involved in adaptive (AID) and innate (APOBEC3s) immunity, lipid metabolism (APOBEC1), and possibly even active DNA demethylation [49,50,51] both in developing and in terminally differentiated cells. Extremely precise, tight, and complex (and therefore, not surprisingly, poorly understood) regulation of AID/APOBEC proteins ensures that in normal cells, they edit cytosines at very specific sites, such as immunoglobulin genes or viral DNA. However, when the regulatory constraints fail, these housekeepers can become much more promiscuous and edit DNA genome-wide.

The overexpression of active APOBECs is highly toxic in human cell lines [18,52], indicating that a precise balance of deaminase production and other factors is required in order to cause non-lethal genome-wide hypermutagenesis and kataegis. This is apparently also true in the case of APOBECs, where only a small fraction of cells with unfettered deaminases and a fine-tuned environment survive and give rise to malignant clones. It is also possible that the sudden overproduction of deaminases in tumor cells with genomes shaped by other mutagenic processes will kill the tumor by extensively damaging its genome, unless the tumor cells can protect themselves against APOBEC.

## 4. Materials and Methods

### 4.1. Mutations in Yeast Genomes

Coordinates and types of mutations induced by various APOBEC/AID proteins in yeast were obtained from previously published SNV datasets (see legend to Supplementary Table S1) [37,53,54,55,56]. To extract the sequence context of the mutations, we used the getfasta tool from the bedtools package (http://bedtools.readthedocs.org/en/latest/). These datasets are available upon request from I.B.R. The logo description of mutable motifs was constructed using the Weblogo website (http://weblogo.berkeley.edu/logo.cgi).

### 4.2. Analysis of Mutable Motifs

Several approaches have been developed for the analysis of a set of mutated sequences [27,28,29]. A mononucleotide weight matrix is a simple and straightforward way to present the structure of a functional signal and to calculate weights for the signal sequence. Each matrix includes information on a normalized frequency of A, T, G, C bases in each of the 10 positions surrounding the detected sites of mutation (5 bases downstream and 5 bases upstream). We calculated the weight matrices for 6f different AID/APOBEC mutational signatures in the yeast genome (Supplementary Table S1). 

A simple formula for W(b,j) was used for data analysis: W(b,j) = log2[f(b,j)/e(b)], where f(b,j) is the observed frequency of the nucleotide b in position j and e(b,j) is the expected frequency of the nucleotide b in position j, calculated as the mean nucleotide frequencies of positions −5,−4, +4, +5 for sites of mutations in the yeast genome; the resulting W(b,i) matrices are shown in Supplementary Figure S1.

The matching score S(b1, ..., bL) of a sequence b1, ..., bL is as follows:

       L S(b1, ..., bL) = Σ W(b,j)       j = 1(1)

The matching score between sequence b1, ..., bL and a weight matrix can be further expressed as a percentage:

% matching score = 100 × (S(b1, ..., bL) − Smin)/(Smax − Smin)(2)

      L                                                         L                    Smin = Σ MIN W(b,j)                       Smax = Σ MAX W(b,j)         j = 1 b                                                  j = 1 b(3)

Hereafter, we use the term “weight” instead of “% matching score”. We used the positions −3:+3 to estimate the weights of the sites.

In addition to the analyses of AID/APOBEC mutational signatures in cancer genomes, we performed a control experiment: we randomly shuffled a dataset of AID/APOBEC contexts in the yeast genome (Supplementary Table S1), keeping position 6 (the position of mutations) intact. Each sequence was shuffled separately; thus, the overall base composition and the base compositions of each sequence were the same. We also performed another control experiment: we randomly extracted sequences from the yeast genome, maintaining the nucleotide composition and the size of sequence sets for each set of mutation sites with AID/APOBEC-induced mutations. Weight matrices were derived from these sampled sites. Where there was a significant difference between an extracted set and the analyzed set (the 2-tailed *t*-test), the sampling procedure was repeated.

### 4.3. Datasets and Analysis of Somatic Mutations

Somatic mutation data from the ICGC and TCGA cancer genome projects were extracted from the Sanger COSMIC Whole Genome Project v75 (http://cancer.sanger.ac.uk/wgs). The ICGC/TCGA datasets are almost exclusively passenger mutations, and they are unlikely to be subject to selection to promote cellular proliferation. Thus, they are more likely to reflect the original AID/APOBEC mutational spectrum [23]. The tissues and cancer types were defined according to the primary tumor site and the cancer project in question [12,13]. A dataset of somatic mutations in mitochondrial DNA in various cancer types was extracted from [32]. In this set, no excess of mutations in known mutable motifs is to be expected, because the mutation landscape in mitochondrial DNA is shaped by its very specific mode of replication [32]. The mitochondrial mutation set can, therefore, be used as a negative control.

DNA sequences surrounding the mutated nucleotide represent the mutation context. We compared the frequency of known mutable motifs for somatic mutations with the frequency of these motifs in the vicinity of the mutated nucleotide. Specifically, for each base substitution, the 121 bp sequence centered at the mutation was extracted (the DNA neighborhood). We used only the nucleotides immediately flanking the mutations, because the AID/APOBEC enzymes are thought to scan a very limited region of DNA to deaminate (methyl)cytosines in a preferred motif [16,57,58]. This approach does not exclude any specific area of the genome, but rather uses the areas within each sample where mutagenesis has occurred (taking into account the variability in the mutation rates across the human genome) and then evaluates whether the mutagenesis in these samples were enriched for AID/APOBEC motifs [58]. This approach was thoroughly tested, and the high accuracy of the analysis was demonstrated [58]. The mean weight of the mutable motifs (Supplementary Figure S1) in the positions of somatic mutations was compared to the mean weight of the same motifs in the DNA neighborhood using the *t*-test (2-tail test) and Monte Carlo test (MC, 1-tail test) similar to the consensus method, as previously described [58].

### 4.4. Impact of AID/APOBEC Mutagenesis

In order to estimate the proportion of mutated sites that are likely to be caused by the AID/APOBEC enzymes, we applied a mixture model of two normal distributions [43] to distributions of weights of somatic mutation contexts. An example of such a distribution is shown in Figure 5B. This approach is based on the method of estimating the protein coding density in a corpus of DNA sequence data, in which a ‘protein-coding coding statistic’ (which is similar to distributions of weights of somatic mutation contexts) is calculated for a large number of windows of the sequence under study. The distribution of the statistic is decomposed into two normal distributions and assumed to be distributions of the coding statistic in the coding and non-coding fractions of the sequence windows [43]. The distribution with the largest mean was assumed to reflect the fraction of protein coding fragments [43]. Similarly, the fraction of sites in a distribution with the largest mean was assumed to be the fraction of mutations induced by the AID/APOBEC enzymes. The results were considered to be reliable only if no significant difference was found between the observed and expected distributions according to the χ^2^ test. The suggested classification approach for normal distributions had been tested by Fickett and Guigo and showed good accuracy [43]. All the details of the suggested methodology and underlined statistical Bayesian framework were previously described for analyses of normal and binomial distributions [43,44].

Heatmap visualization analysis for each of the AID/APOBEC pseudo-mutable motifs groups was performed. The R (https://www.R-project.org/) software package heatmap.2 (https://CRAN.R-project.org/package=gplots) was employed to generate the heatmaps for each group. For each group, a specific range of values was established in grayscale representation, from the lowest values to the highest values. For the pseudo-mutable motifs in somatic mutation in the C:G sites group, the range was from 0.01 to 0.84 with intervals between 0.05. Values <0.01 were denoted as white. For the pseudo-mutable motifs in somatic mutation in the C:G>T:A sites group, the range was from 1 to 1.573 with intervals between 0.01. Values <1 were denoted as white. For the pseudo-mutable motifs in somatic mutation in the C:G>C:G sites group, the range was from 1 to 1.802 with intervals between 0.01. Values <1 were denoted as white. For the pseudo-mutable motifs in somatic mutation in the C:G>A:T sites group, the range was from 1 to 1.362 with intervals between 0.02. Values <1 were denoted as white.

### 4.5. Control Experiment 1: Analysis of Somatic Mutations in Mitochondrial DNA

In the first control experiment, we analyzed the sequence context of somatic mutations in mitochondrial DNA. In this set, no excess of mutations in known mutable motifs was to be expected, because the mutation landscape in mitochondrial DNA is shaped by its very specific mode of replication [32]. Thus, the mitochondrial mutation set can be used as a negative control. No significant excess of AID/APOBEC mutable motifs was found (Supplementary Table S2). This is consistent with a previous study [32]. In all the studied tissues, the ratio of the mean weight of the mutated sites vs. the mean weight of the non-mutated sites was less than or close to 1; this is expected when there is no correlation between mutable motifs and mutation (Supplementary Table S2). We observed only a single case where the Monte Carlo test yielded a significant P-value (*P* = 0.031, APOBEC3B/brain), but this result was not confirmed by use of the *t*-test and is likely to be an isolated false positive. Thus, the weight matrix appears to be a reliable method for the analysis of somatic mutations.

### 4.6. Control Experiment 2: Correlation between the Matrices of Shuffled Sites of Mutations and the Sites of Somatic Mutation in Cancer Cells

In order to allow for differences in nucleotide content between the yeast and human genomes, we used normalized weight matrices (see above). To test the robustness of the normalization, a simple control experiment was designed: we randomly shuffled the sequences of the AID/APOBEC mutation sites (Supplementary Table S1). We identified rare cases of a significant deviation from the expected value of the ratio (1.0, the ratio is the mean weight of the mutated sites divided by the mean weight of the non-mutated sites), but those cases constituted only 2.6% of all the studied cases (Supplementary Table S3). This result establishes that the weight matrix technique yields an expected proportion of false positives (the expected false discovery rate should be around 5% according to the standard in the field [33]) and hence is robust with respect to the biased nucleotide composition of mutated sites in the yeast genome. However, the results for colon, skin, and stomach cancers may not be reliable for some APOBECs (the fractions of false positives were found to be large, for example, 0.96 for APOBEC3C/skin; Supplementary Table S3). In general, the APOBEC3C weight matrix tends to yield the largest number of false positives, suggesting that this matrix might be problematic. We conclude that such controls should always be performed when starting work with a new mutation set. 

### 4.7. Control Experiment 3: Correlation between Matrices of Randomly Sampled Sites from the Yeast Genome and Somatic Mutations in Cancer Cells

**Figure 6:**
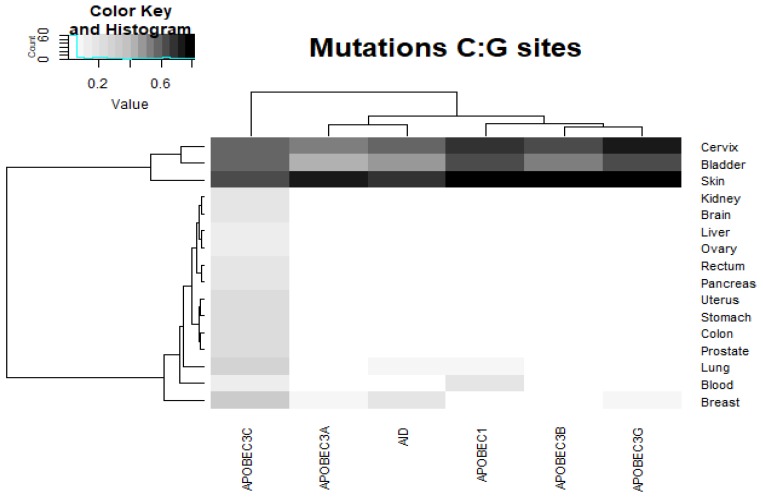
Fraction of random matrices with a significant correlation between AID/APOBEC pseudo-mutable motifs (randomly sampled sites from the yeast genome) and the sequence context of somatic mutations in C:G sites. For the actual data, see Supplementary Table S2. The intensities of the gray color correspond to the fractions of cases with a significant correlation between pseudo-mutable motifs (represented as weight matrices) and the context of somatic mutations in C:G sites.

To check for a potential influence of nucleotide content biases and the extent of a correlation between positions in yeast and human genomes, we randomly extracted sequences from the yeast genome, maintaining the nucleotide composition and size of sequence sets for each set of mutation sites. Weight matrices were derived from these sampled sites. We identified numerous examples of a substantial deviation from the expected values that produced significant results that should be considered to be false positives (Figure 6 and Supplementary Table S4), because we did not expect any meaningful association between randomly sampled sites and somatic mutations. The APOBEC3C weight matrix yielded a large number of significant yet spurious results (false positives) for all the studied tissues (Figure 6 and Supplementary Table S4) and therefore cannot be recommended for the analysis of somatic mutation. This effect may have been due to the much smaller number of mutations in the dataset, a lack of highly informative positions and a high A/T content of sites for APOBEC3C (Figure 1). The results for APOBEC3C are likely to be false positives in this and previous control experiments and were included in the Supplementary Materials only. 

The analysis of mutations in various tissues suggested that the weight matrix technique may also produce misleading results for bladder, cervix, and skin tumors (Figure 6). The skin tissue consistently produced a high rate of false positives in control experiments 3 and 4; thus, weight matrices should be used with great caution for this tissue. The analysis of nucleotide frequencies for the region ±3 suggested that skin, cervix, and bladder tumors are characterized by a high frequency of T nucleotides around the sites of mutation (Supplementary Table S5), and this is likely to be a reason for the high rate of false positives. It should be noted that other techniques are also likely to produce a high rate of false positives for these tissues, although this type of control experiment has, to our knowledge, never been performed before except for analysis of somatic mutations in normal tissues [21]. The likely reason for high rates of false positives is that APOBEC mutable motifs tend to be A/T-rich (even C-rich APOBEC3G sites contain excessive amounts of A and T nucleotides in positions −3, +1, +2, and +3; Figure 1 and Supplementary Figure S1). We attempted to take this into account by removing sites with a high A/T content (≥50% A+T in the 10-nucleotide region around sites of somatic mutations, Supplementary Table S4). Although there was a substantial improvement in the accuracy of prediction (rates of false positives were much smaller, Supplementary Table S4), problems with the accuracy of prediction for skin tumors persisted (Supplementary Table S4).

### 4.8. Control Experiment 4: Analysis of Somatic Mutations in Human Immunoglobulin Genes

Somatic mutations in human immunoglobulin genes are known to be associated with AID mutable motifs [35], and these mutations can be used as a positive control set. Indeed, a significant association between the AID mutable motif and mutations was found in all three studied sets of somatic mutations [34,35] (Table 3), suggesting that the AID weight matrix is a reliable descriptor of AID-induced mutagenesis. The APOBEC1/3A/3B/3G weight matrices did not, however, yield significant results for all the studied cases (Table 3). This is consistent with the absence of any traces of APOBEC1/3A/3B/3G-induced mutation in the somatic hypermutation profiles of immunoglobulin genes [36]. The results of all four control experiments suggested that the weight matrix technique is applicable to studied APOBECs.

## 5. Conclusions

For the first time, we have adopted the weight matrix (sequence profile) approach for the analysis of mutations in cancer genomes, and we provide evidence for this method being a more precise descriptor of mutations than the commonly used sequence consensus approach. Control experiments using shuffled sites and constrained samples of randomly sampled sequences from the yeast genome yielded a low level of false positives. 

We confirm that while mutational footprints of APOBEC1, APOBEC3A, APOBEC3B, and APOBEC3G are prominent in many cancers, mutable motifs characteristic of the action of the humoral immune response somatic hypermutation enzyme, AID, are the most widespread feature of the somatic mutation spectra attributed to APOBECs in cancer genomes. The AID and APOBEC3A mutable motifs are the most prominent features of the C:G>T:A transitions that constitute the vast majority of somatic mutations in studied cancers. We also demonstrated an abundance of APOBEC3A/3B/3G mutable motifs in DNA contexts of C:G>A:T transversions. A potential association of AID and APOBEC3A in a certain type of blood cancers is another interesting outcome of our study. Overall, the weight matrix approach revealed that somatic mutations are significantly associated with at least one AID/APOBEC mutable motif in the studied cancer types.

## Figures and Tables

**Table 1:** Pairwise differences between the DNA context (position-specific nucleotide frequencies across ±5 surrounding bases) of the studied AID/APOBEC proteins.

	AID	APOBEC3G	APOBEC3C	APOBEC3B	APOBEC3A
**APOBEC1**	1986.8	2299.2	203.2	378.6	344.1
**APOBEC3A**	1674.4	2057.0	138.4	175.7	
**APOBEC3B**	1764.5	2316.8	175.7		
**APOBEC3C**	237.2	327.5			
**APOBEC3G**	2711.8				

The critical χ^2^ values = 71.1 (after Bonferroni correction *P* = 0.05/15 = 0.0033, degrees of freedom = 42). The χ^2^ test was applied to raw numbers of nucleotides.

**Table 2:** Correlation between AID/APOBEC mutable motifs and the context of somatic mutations in C:G sites in various blood and skin tumor types.

Cancer Tissue Type	Number of Mutations	Test	APOBEC1	APOBEC3A	APOBEC3B	APOBEC3G	AID
Blood: acute myeloid leukemia	6844	Ratio	0.920	0.978	0.958	0.977	1.031
		*t*-test	NSE #	NSE	NSE	NSE	6.5 *
		MC test					<0.001
		Fraction					
Blood: GCB lymphomas	2747	Ratio	0.967	1.030	0.979	0.980	1.091
		*t*-test	NSE	3.4 *	NSE	NSE	12.3 *
		MC test		<0.001			<0.001
		Fraction		0.208			
Skin: cutaneous melanoma	235043	Ratio	1.388	1.308	1.334	1.138	1.026
		*t*-test	321.3 *	292.8 *	344.6*	176.2 *	35.8 *
		MC test	<0.001	<0.001	<0.001	<0.001	<0.001
		Fraction	0.608		0.508	0.982	0.687
Skin: adeno-carcinoma	780	Ratio	1.045	1.073	1.088	1.075	1.025
		*t*-test	NSE	4.4 *	4.8 *	4.6 *	NSE
		MC test		<0.001	<0.001	<0.001	
		Fraction		0.213			

#—NSE (no significant excess) indicates the absence of a significant excess of mutations in the mutable motifs, suggesting that there is no association between mutagenesis and the motifs. The significance of any excess was measured using the Student *t* and Monte Carlo (MC) tests. The bold font and asterisk (*) denote that the corresponding *P* < 0.002 (critical value = 3.1); this is a conservative estimate of the critical overall value of the *t*-test having allowed for multiple testing by means of the Bonferroni correction (4 × 6 = 24). The “Ratio” is the mean weight of the mutated sites divided by the mean weight of the non-mutated sites. The predicted fraction of mutations induced by AID/APOBEC proteins (“Fraction”) is shown when a significant excess of somatic mutations in the mutable motif comparisons was detected; all cases where there was a significant difference between the observed and expected distributions (*P* > 0.05) were discarded.

**Table 3:** Correlation between the AID/APOBEC mutable motifs and the sequence context of somatic mutations in fragments of human immunoglobulin genes.

Locus	Number of Mutations	Test	APOBEC1	APOBEC3A	APOBEC3B	APOBEC3G	AID
V_H_26	708	Ratio	0.931	0.986	0.919	0.908	1.162
		*t*-test	NSE #	NSE	NSE	NSE	11.1 *
		MC test					<0.001
		Fraction					0.477
J_H_4 intron, control individuals	177	Ratio	0.927	0.957	0.887	0.870	1.331
		*t*-test	NSE	NSE	NSE	NSE	11.9 *
		MC test					<0.001
		Fraction					0.559
J_H_4 intron, XP-V patients	235	Ratio	0.981	1.008	0.957	0.930	1.266
		*t*-test	NSE	NSE	NSE	NSE	9.6 *
		MC test					<0.001
		Fraction					0.366

#—NSE (no significant excess) indicates the absence of a significant excess of mutations in mutable motifs, suggesting that there is no association between mutagenesis and the motifs. The significance of any excess was measured using the Student *t* and Monte Carlo (MC) tests. The bold font and asterisk (*) denote that the corresponding *P* < 0.003 (critical value = 3.1); this is a conservative estimate of the critical overall value of the *t*-test having allowed for multiple testing by means of the Bonferroni correction (3 × 6 = 18). The “Ratio” is the mean weight of the mutated sites divided by the mean weight of the non-mutated sites. The predicted fraction of mutations induced by the AID/APOBEC proteins (“Fraction”) is shown when a significant excess of somatic mutations in the mutable motif comparisons was detected; all the cases where there was a significant difference between the observed and expected distributions (*P* > 0.05) were discarded.

## Supplementary Materials

The following are available online at https://www.mdpi.com/2072-6694/11/2/211/s1, Figure S1:AID/APOBEC weight matrices W(b,j).itle; Figure S2: The overall distribution of fraction of somatic C:G>T:A mutations associated with AID/APOBEC deamination (APOBEC1, APOBEC3A, ABOPEC3B, and AID deaminases); Table S1: Datasets of mutations induced by overexpression of AID/APOBEC enzymes in the yeast genome; Table S2: Control study: correlation between AID/APOBEC mutable motifs and the context of somatic mutations in C:G sites in mitochondrial DNA; Table S3: Control study: fractions of random matrices with a significant correlation between AID/APOBEC pseudo-mutable motifs (shuffled sites of mutations) and the context of somatic mutations at C:G sites; Table S4: Control study: fraction of random matrices with a significant correlation between AID/APOBEC pseudo-mutable motifs (randomly sampled sites from the yeast genome) and the context of somatic mutations at C:G sites; Table S5: Nucleotide composition of the DNA context of somatic mutations (±3 nucleotides); Table S6: Correlation between AID/APOBEC mutable motifs and the context of C:G>T:A somatic mutations; Table S7: Correlation between AID/APOBEC mutable motifs and the context of C:G>G:C somatic mutations; and Table S8: Correlation between AID/APOBEC mutable motifs and the context of C:G>A:T somatic mutations.
