# Supplementary material for: Nucleotide Weight Matrices Reveal Ubiquitous Mutational Footprints of AID/APOBEC Deaminases in Human Cancer Genomes
Source: Cancers (Basel). 2019 Feb 12;11(2):211. doi: 10.3390/cancers11020211 (PMC6406962; doi:10.3390/cancers11020211)

Supplementary Materials

Nucleotide weight matrices reveal ubiquitous mutational footprints of AID/APOBEC deaminases in human cancer genomes

Igor B. Rogozin, Abiel Roche-Lima, Artem G. Lada, Frida Belinky, Ivan A. Sidorenko, Galina V. Glazko, Vladimir N. Babenko, David N. Cooper, Youri I. Pavlov

Abbreviations: APO1 = APOBEC1, APO3A = APOBEC3A, APO3B = APOBEC3B, APO3C = APOBEC3C, APO3G = APOBEC3G, AID = Activation Induced Deaminase

Supplementary Table S1. Datasets of mutations induced by over-expression of AID/APOBEC enzymes in the yeast genome.

Enzyme	Number of mutations (sites)	Lada et al., 2011	Taylor et al., 2013	Lada et al., 2015	Lada et al., 2017
APO1	673	-	+	+	+
APO3A	723	-	+	-	-
APO3B	745	-	+	-	-
APO3C	67	-	+	-	-
APO3G	1147	+	+	+	+
AID	397	-	+	+	+

References:

Lada A.G., Krick C.F., Kozmin S.G., Mayorov V.I., Karpova T.S., Rogozin I.B., Pavlov Y.I. (2011) Mutator effects and mutation signatures of editing deaminases produced in bacteria and yeast. Biochemistry (Mosc.), 76: 131-146.

Taylor BJ, Nik-Zainal S, Wu YL, Stebbings LA, Raine K, Campbell PJ, Rada C, Stratton MR, Neuberger MS. (2013) DNA deaminases induce break-associated mutation showers with implication of APOBEC3B and 3A in breast cancer kataegis. Elife 2: e00534.

Lada A.G., Kliver S.F., Dhar A., Polev D., Masharsky A., Rogozin I.B., Pavlov Y.I. (2015) Disruption of transcriptional coactivator Sub1 leads to genome-wide re-distribution of clustered mutations induced by APOBEC in active yeast genes. PLoS Genet. 11: e1005217.

Lada A.G., Stepchenkova E.I., Zhuk A.S., Kliver S.F., Rogozin I.B., Polev D., Dhar A., Pavlov Y.I. (2017) Recombination is responsible for the increased recovery of drug-resistant mutants with hypermutated genomes in resting yeast diploids expressing APOBEC deaminases. Front. Genet. 8: 202.

Supplementary Table S2. Control study: correlation between AID/APOBEC mutable motifs and the context of somatic mutations in C:G sites in mitochondrial DNA.

Cancer tissue type	#Muta-tions	Test	APO1	APO3A	APO3B	APO3C	APO3G	AID
Bladder	39	Ratio<br>*t*-test<br>MC test	.912<br>NSE	.913<br>NSE	.908<br>NSE	.958<br>NSE	.897<br>NSE	.937<br>NSE
Blood	66	Ratio<br>*t*-test<br>MC test	.887<br>NSE	.917<br>NSE	.907<br>NSE	.902<br>NSE	.912<br>NSE	.901<br>NSE
Brain	4	Ratio<br>*t*-test<br>MC test	.901<br>NSE	1.018<br>NSE<br>0.089	1.028<br>NSE<br>0.031	.923<br>NSE	.985<br>NSE	1.007<br>NSE<br>0.342
Breast	283	Ratio<br>*t*-test<br>MC test	.918<br>NSE	.932<br>NSE	.925<br>NSE	.946<br>NSE	.932<br>NSE	.912<br>NSE
Cervix	22	Ratio<br>*t*-test<br>MC test	.935<br>NSE	.901<br>NSE	.927<br>NSE	.946<br>NSE	.866<br>NSE	.875<br>NSE
Colon	81	Ratio<br>*t*-test<br>MC test	.892<br>NSE	.904<br>NSE	.898<br>NSE	.913<br>NSE	.886<br>NSE	.906<br>NSE
Kidney	7	Ratio<br>*t*-test<br>MC test	.917<br>NSE	.864<br>NSE	.911<br>NSE	.971<br>NSE	.931<br>NSE	.901<br>NSE
Liver	49	Ratio<br>*t*-test<br>MC test	.818<br>NSE	.849<br>NSE	.828<br>NSE	.880<br>NSE	.833<br>NSE	.881<br>NSE
Lung	55	Ratio<br>*t*-test<br>MC test	.930<br>NSE	.954<br>NSE	.929<br>NSE	.976<br>NSE	.987<br>NSE	.904<br>NSE
Ovary	34	Ratio<br>*t*-test<br>MC test	.889<br>NSE	.883<br>NSE	.858<br>NSE	.923<br>NSE	.827<br>NSE	.857<br>NSE
Prostate	79	Ratio<br>*t*-test<br>MC test	.927<br>NSE	.916<br>NSE	.930<br>NSE	.965<br>NSE	.938<br>NSE	.937<br>NSE

Skin	16	Ratio<br>*t*-test<br>MC test	.867<br>NSE	.836<br>NSE	.871<br>NSE	.904<br>NSE	.878<br>NSE	.901<br>NSE
Stomach	23	Ratio<br>*t*-test<br>MC test	.824<br>NSE	.867<br>NSE	.873<br>NSE	.939<br>NSE	.947<br>NSE	.938<br>NSE
Uterus	40	Ratio<br>*t*-test<br>MC test	.966<br>NSE	.979<br>NSE	.972<br>NSE	.946<br>NSE	.971<br>NSE	.867<br>NSE

NSE indicates absence of significant excess of mutations in AID/APOBEC mutable motifs (NSE, no significant excess) suggesting that there is no association between mutagenesis and motifs. The correlation was measured using Student *t*-test and Monte Carlo (MC) tests. The asterisk denotes that the corresponding $P < 0.0005$ (critical value = 3.291) is a conservative estimate of the critical overall value of the *t*-test taking into account the Bonferroni correction for multiple testing (16 x 6 = 96). "Ratio" is the mean weight of mutated sites divided by the mean weight of non-mutated sites. Results of the MC test are shown for cases where the Ratio > 1 (shown in green).

Supplementary Table S3. Control study: fractions of random matrices with a significant correlation between AID/APOBEC pseudo-mutable motifs (shuffled sites of mutations) and the context of somatic mutations [IGOR: at?] C:G sites.

Cancer tissue type	#Mutations	APO1	APO3A	APO3B	APO3C	APO3G	AID
Bladder	38750	0.	0.	0.	0.	0.	0.
Blood	10633	0.	0.	0.	0.	0.	0.
Brain	30926	0.	0.	0.	0.04	0.	0.03
Breast	48035	0.	0.	0.	0.	0.	0.
Cervix	41454	0.	0.	0.	0.	0.	0.
Colon	175109	0.	0.03	**0.12**	**0.21**	0.	**0.35**
Kidney	32382	0.	0.	0.	0.	0.	0.
Liver	74161	0.	0.	0.	0.01	0.	0.
Lung	180284	0.	0.	0.	0.	0.	0.
Ovary	22340	0.	0.	0.	0.	0.	0.
Pancreas	35165	0.	0.	0.	0.03	0.	0.
Prostate	16703	0.	0.	0.	**0.30**	0.	0.
Rectum	30018	0.	0.03	**0.18**	0.	0.	**0.17**
Skin	244248	**0.81**	**0.33**	0.	**0.96**	0.	0.
Stomach	115652	0.	0.	**0.15**	**0.15**	0.	**0.19**
Uterus	55999	0.	0.	0.	0.09	0.	0.

The significance of the excess of somatic mutations observed in a pseudo-mutable context was measured using the Student *t*-test and Monte Carlo (MC) tests (see Materials and Methods for details). The threshold value was corrected using the conservative Bonferroni correction for multiple testing (16 tissues x 6 mutable contexts = 96, $P < 0.0005$, critical t-test value = 3.291). The expected false discovery rate should be around 0.05, large fraction values (>0.10) are shown in green.

Supplementary Table S4. Control study: fraction of random matrices with a significant correlation between AID/APOBEC pseudo-mutable motifs (randomly sampled sites from the yeast genome) and the context of somatic mutations at C:G sites.

Cancer tissue type	#Mutations	APO1	APO3A	APO3B	APO3C	APO3G	AID
All somatic mutations							
Bladder	38750	**0.65**	**0.46**	**0.56**	**0.57**	**0.64**	**0.50**
Blood	10633	**0.19**	0.04	0.	**0.16**	0.01	0.
Brain	30926	0.	0.02	0.	**0.17**	0.01	0.01
Breast	48035	0.	0.07	0.	**0.35**	**0.10**	**0.21**
Cervix	41454	**0.70**	**0.56**	**0.64**	**0.58**	**0.73**	**0.61**
Colon	175109	0.	0.04	0.02	**0.25**	0.	0.
Kidney	32382	0.	0.02	0.01	**0.18**	0.01	0.02
Liver	74161	0.	0.04	0.01	**0.16**	0.01	0.
Lung	180284	0.07	0.02	0.04	**0.29**	0.01	0.09
Ovary	22340	0.	0.02	0.01	**0.15**	0.01	0.
Pancreas	35165	0.01	0.04	0.02	**0.20**	0.01	0.01
Prostate	16703	0.01	0.03	0.02	**0.23**	0.01	0.
Rectum	30018	0.01	0.04	0.02	**0.19**	0.02	0.02
Skin	244248	**0.79**	**0.72**	**0.80**	**0.63**	**0.84**	**0.70**
Stomach	115652	0.	0.04	0.01	**0.24**	0.01	0.03
Uterus	55999	0.01	0.04	0.02	**0.22**	0.02	0.02
Somatic mutations with a C:G-rich DNA context (≥50% C+G in the 10 nucleotide region around sites of somatic mutations)							
Bladder	38750	**0.42**	**0.28**	**0.30**	**0.47**	**0.32**	**0.41**
Cervix	41454	**0.46**	**0.29**	**0.37**	**0.40**	**0.39**	**0.48**
Skin	244248	**0.74**	**0.66**	**0.71**	**0.57**	**0.75**	**0.71**

The significance of the excess of somatic mutations observed in a pseudo-mutable context was measured using the Student *t*-test and Monte Carlo (MC) tests (see Materials and Methods for details). The threshold value was corrected using the conservative Bonferroni correction for multiple testing (16 tissues x 6 mutable contexts = 96, $P < 0.0005$, critical t-test value = 3.291). The expected false discovery rate should be around 0.05, large fraction values (>0.10) are shown in green.

Supplementary Table S5. Nucleotide composition of the DNA context of somatic mutations (±3 nucleotides).

	A	T	G	C
**Bladder**	**.212**	**.328**	**.212**	**.248**
Blood	.230	.224	.294	.252
Brain	.224	.213	.311	.252
Breast	.221	.300	.234	.245
**Cervix**	**.206**	**.350**	**.200**	**.244**
Colon	.196	.239	.328	.237
Kidney	.231	.255	.251	.263
Liver	.215	.236	.268	.281
Lung	.222	.254	.246	.278
Ovary	.234	.244	.262	.259
Pancreas	.214	.225	.309	.252
Prostate	.207	.215	.296	.281
Rectum	.207	.278	.294	.221
**Skin**	**.177**	**.356**	**.175**	**.293**
Stomach	.198	.219	.342	.242
Uterus	.209	.269	.306	.217

Sites with mutations in G nucleotides were converted to the complementary strand

Supplementary Table S6. Correlation between AID/APOBEC mutable motifs and the context of C:G > T:A somatic mutations.

Cancer tissue type	#Muta-tions	Test	APO1	APO3A	APO3B	APO3C	APO3G	AID
Bladder	23127	Ratio	1.487*	1.463*	1.461*	1.179*	1.101*	1.051*
		t-test	112.845	134.038	120.517	71.132	41.798	21.415
		MC test	<0.001	<0.001	<0.001	<0.001	<0.001	<0.001
		Fraction	.657		.627	.138	.921	.500
Blood	6331	Ratio	.946	1.026*	.978	1.021*	1.009	1.065*
		t-test	NSE	4.330	NSE	4.356	NSE	12.015
		MC test		<0.001		<0.001		<0.001
		Fraction		.190		.195		
Brain	23354	Ratio	.855	.975	.888	.992	.960	1.063*
		t-test	NSE	NSE	NSE	NSE	NSE	21.831
		MC test						<0.001
		Fraction						
Breast	10327	Ratio	1.233*	1.261*	1.231*	1.098*	1.039*	1.050*
		t-test	34.183	48.309	37.605	25.377	9.677	12.992
		MC test	<0.001	<0.001	<0.001	<0.001	<0.001	<0.001
		Fraction	.476			.123	.866	
Cervix	23034	Ratio	1.573*	1.542*	1.538*1	1.207*	1.113*	1.060*
		t-test	138.455	168.841	50.422	82.775	49.688	25.686
		MC test	<0.001	<0.001	<0.001	<0.001	<0.001	<0.001
		Fraction	.737		.707	.165	.165	.339
Colon	132513	Ratio	.838	1.003	.893	.989	.916	1.080*
		t-test	NSE	NSE	NSE	NSE	NSE	65.985
		MC test						<0.001
		Fraction						
Kidney	16690	Ratio	.972	1.024*	.995	1.020*	.989	1.039*
		t-test	NSE	6.486	NSE	6.899	NSE	11.814
		MC test		<0.001		<0.001		<0.001
		Fraction		.230		.215		
Liver	42221	Ratio	.915	.968	.946	.997	.998	1.014*
		t-test	NSE	NSE	NSE	NSE	NSE	6.400
		MC test						<0.001
		Fraction						
Lung	58116	Ratio	1.164*	1.179*	1.179*	1.077*	1.078*	1.014*
		t-test	59.858	82.536	72.640	48.475	42.941	8.518
		MC test	<0.001	<0.001	<0.001	<0.001	<0.001	<0.001
		Fraction	.393			.091	.815	
Ovary	10973	Ratio	.960	1.031*	.990	1.012*	.992	1.041*
		t-test	NSE	6.851	NSE	3.355	NSE	9.813
		MC test		<0.001		<0.001		<0.001
		Fraction		.199		.205		
Pancreas	25544	Ratio	.921	1.039*	.966	1.010*	.977	1.058*
		t-test	NSE	13.290	NSE	4.034	NSE	21.396
		MC test		<0.001		<0.001		<0.001
		Fraction		.178		.291		

Prostate	9506	Ratio<br>*t*-test<br>MC test<br>Fraction	.866<br>NSE	1.001<br>NSE	.910<br>NSE	1.013*<br>3.411<br><0.001<br>.230	.962<br>NSE	1.082*<br>18.442<br><0.001
Rectum	20315	Ratio<br>*t*-test<br>MC test<br>Fraction	.956<br>NSE	1.086*<br>24.646<br><0.001<br>.239	1.009<br>NSE	1.028*<br>10.270<br><0.001	.965<br>NSE	1.076*<br>25.245<br><0.001
Skin	230979	Ratio<br>*t*-test<br>MC test<br>Fraction	1.438*<br>331.007<br><0.001<br>.617	1.333*3<br>00.669<br><0.001	1.402*3<br>69.514<br><0.001<br>.527	1.189*2<br>23.837<br><0.001<br>.281	1.148*<br>178.865<br><0.001<br>.980	1.038*<br>46.377<br><0.001<br>.667
Stomach	92882	Ratio<br>*t*-test<br>MC test<br>Fraction	.799<br>NSE	.970<br>NSE	.859<br>NSE	.970<br>NSE	.901<br>NSE	1.070*<br>48.316<br><0.001
Uterus	41875	Ratio<br>*t*-test<br>MC test<br>Fraction	.931<br>NSE	1.089*<br>35.553<br><0.001<br>.292	.980<br>NSE	1.046*<br>23.344<br><0.001	.929<br>NSE	1.107*<br>52.688<br><0.001

NSE means that No Significant Excess of mutable motifs was found. The significance of the observed excess was measured using the Student *t*-test and Monte Carlo (MC) tests. The asterisk denotes that the corresponding $P < 0.0005$ (critical value = 3.291) is a conservative estimate of the critical overall value of the *t*-test taking into account the Bonferroni correction for multiple testing (16 x 6 = 96). “Ratio” is the mean weight of mutated sites divided by the mean weight of non-mutated sites. The predicted fraction of mutations induced by AID/APOBEC proteins (“Fraction”) is shown when the significant excess of somatic mutations in a mutable motif comparisons was detected; all cases where there was a significant difference between observed and expected distributions ($P > 0.05$) were discarded.

Supplementary Table S7. Correlation between AID/APOBEC mutable motifs and the context of C:G > G:C somatic mutations.

Cancer tissue type	#Muta-tions	Test	APO1	APO3A	APO3B	APO3C	APO3G	AID
Bladder	11020	Ratio<br>*t*-test<br>MC test<br>Fraction	1.698*<br>146.887<br><0.001	1.575*<br>149.530<br><0.001<br>.795	1.614*1<br>53.222<br><0.001<br>.819	1.178*<br>58.002<br><0.001<br>.758	1.162*<br>55.175<br><0.001<br>.266	1.015*<br>4.832<br><0.001
Blood	2095	Ratio<br>*t*-test<br>MC test<br>Fraction	.911<br>NSE	.939<br>NSE	.922<br>NSE	.936<br>NSE	.903<br>NSE	1.006<br>NSE
Brain	3306	Ratio<br>*t*-test<br>MC test<br>Fraction	1.160*<br>14.417<br><0.001<br>.394	1.101*<br>11.126<br><0.001<br>.363	1.126*<br>11.777<br><0.001<br><0.001	1.036*<br>5.912<br><0.001<br>.147	1.013<br>NSE	1.033*<br>4.534<br><0.001<br>.550
Breast	22746	Ratio<br>*t*-test<br>MC test<br>Fraction	1.553*<br>143.860<br><0.001	1.451*<br>141.757<br><0.001<br>.718	1.487*<br>141.726<br><0.001<br>.733	1.138*<br>61.792<br><0.001<br>.109	1.111*<br>49.665<br><0.001<br>.193	1.019*<br>8.114<br><0.001<br>.234
Cervix	12993	Ratio<br>*t*-test<br>MC test<br>Fraction	1.802*<br>224.947<br><0.001	1.667*<br>228.869<br><0.001<br>.872	1.705*<br>237.999<br><0.001<br>.915	1.214*<br>76.699<br><0.001<br>.648	1.174*<br>71.539<br><0.001<br>.308	1.033*<br>11.911<br><0.001
Colon	6336	Ratio<br>*t*-test<br>MC test<br>Fraction	1.080*<br>9.495<br><0.001	1.032*<br>4.781<br><0.001<br>.321	1.039*<br>5.040<br><0.001	.988<br>NSE	.949<br>NSE	1.029*<br>5.476<br><0.001
Kidney	5867	Ratio<br>*t*-test<br>MC test<br>Fraction	1.129*<br>15.831<br><0.001<br>.380	1.070*<br>10.701<br><0.001<br>.334	1.114*<br>15.046<br><0.001	1.027*<br>6.015<br><0.001<br>.153	1.043*<br>7.853<br><0.001	1.017<br>NSE
Liver	8314	Ratio<br>*t*-test<br>MC test<br>Fraction	1.142*<br>20.167<br><0.001<br>.368	1.091*<br>16.430<br><0.001<br>.329	1.126*<br>19.542<br><0.001	1.029*<br>7.435<br><0.001<br>.129	1.043*<br>8.894<br><0.001	1.012<br>NSE
Lung	32118	Ratio<br>*t*-test<br>MC test<br>Fraction	1.289*<br>78.368<br><0.001<br>.522	1.250*<br>86.746<br><0.001	1.273*<br>82.654<br><0.001	1.081*<br>41.739<br><0.001<br>.093	1.076*<br>33.698<br><0.001<br>.856	1.032*<br>14.806<br><0.001<br>.359
Ovary	5609	Ratio<br>*t*-test<br>MC test<br>Fraction	1.153*<br>17.649<br><0.001<br>.395	1.105*<br>15.263<br><0.001	1.131*<br>16.282<br><0.001	1.031*<br>6.634<br><0.001<br>.082	1.031*<br>5.451<br><0.001<br>.780	1.022*<br>3.928<br><0.001
Pancreas	3426	Ratio<br>*t*-test<br>MC test<br>Fraction	1.232*<br>20.503<br><0.001<br>.448	1.158*<br>17.298<br><0.001<br>.405	1.195*<br>18.502<br><0.001	1.049*<br>7.899<br><0.001<br>.103	1.032*<br>4.606<br><0.001<br>.716	1.024*<br>3.340<br>0.001

Prostate	1387	Ratio<br>*t*-test<br>MC test<br>Fraction	1.130*<br>7.583<br><0.001<br>.396	1.088*<br>6.648<br><0.001<br>.327	1.106*<br>6.730<br><0.001	1.012<br>NSE	1.042*<br>3.587<br><0.001<br>.839	.994<br>NSE
Rectum	1975	Ratio<br>*t*-test<br>MC test<br>Fraction	1.120*<br>8.026<br><0.001<br>.401	1.086*<br>7.207<br><0.001<br>.368	1.103*<br>7.400<br><0.001<br>.570	1.032*<br>3.960<br><0.001<br>.094	.973<br>NSE	1.055*<br>6.052<br><0.001
Skin	5027	Ratio<br>*t*-test<br>MC test<br>Fraction	1.141*<br>15.681<br><0.001<br>.364	1.099*<br>14.045<br><0.001	1.135*<br>16.676<br><0.001	1.032*<br>6.394<br><0.001<br>.115	1.075*<br>11.708<br><0.001	.999<br>NSE
Stomach	4960	Ratio<br>*t*-test<br>MC test<br>Fraction	1.120*<br>12.627<br><0.001<br>.390	1.061*<br>8.030<br><0.001<br>.349	1.075*<br>8.504<br><0.001<br>.558	.993<br>NSE	.954<br>NSE	1.018<br>NSE
Uterus	2221	Ratio<br>*t*-test<br>MC test<br>Fraction	1.210*<br>14.995<br><0.001<br>.465	1.158*<br>13.702<br><0.001<br>.442	1.163*<br>12.451<br><0.001	1.035*<br>4.621<br><0.001<br>.857	1.011<br>NSE	1.023<br>NSE

NSE means that No Significant Excess of mutable motifs was found. The significance of the observed excess was measured using the Student *t*-test and Monte Carlo (MC) tests. The asterisk denotes that the corresponding $P < 0.0005$ (critical value = 3.291) is a conservative estimate of the critical overall value of the *t*-test taking into account the Bonferroni correction for multiple testing ($16 \times 6 = 96$). "Ratio" is the mean weight of mutated sites divided by the mean weight of non-mutated sites. The predicted fraction of mutations induced by AID/APOBEC proteins ("Fraction") is shown when a significant excess of somatic mutations in a mutable motif comparisons was detected; all cases where there was a significant difference between observed and expected distributions ($P > 0.05$) were discarded.

Supplementary Table S8. Correlation between AID/APOBEC mutable motifs and the context of C:G > A:T somatic mutations.

Cancer tissue type	#Muta-tions	Test	APO1	APO3A	APO3B	APO3C	APO3G	AID
Bladder	4603	Ratio<br>*t*-test<br>MC test<br>Fraction	1.295*<br>29.792<br><0.001<br>.522	1.259*<br>32.106<br><0.001	1.291*<br>32.703<br><0.001<br>.490	1.088*<br>15.633<br><0.001<br>.116	1.045*<br>7.384<br><0.001<br>.892	1.020*<br>3.348<br><0.001<br>.444
Blood	2207	Ratio<br>*t*-test<br>MC test<br>Fraction	.883<br>NSE	.938<br>NSE	.926<br>NSE	.990<br>NSE	.937<br>NSE	1.068*<br>7.287<br><0.001
Brain	4266	Ratio<br>*t*-test<br>MC test<br>Fraction	.995<br>NSE	.995<br>NSE	1.015<br>NSE	1.011<br>NSE	1.004<br>NSE	1.042*<br>6.606<br><0.001
Breast	14962	Ratio<br>*t*-test<br>MC test<br>Fraction	1.146*<br>27.304<br><0.001<br>.411	1.124*<br>28.109<br><0.001<br>.361	1.152*<br>31.022<br><0.001	1.046*<br>15.073<br><0.001<br>.122	1.015*<br>4.222<br><0.001<br>.831	1.019*<br>5.583<br><0.001
Cervix	5427	Ratio<br>*t*-test<br>MC test<br>Fraction	1.358*<br>40.229<br><0.001<br>.594	1.314*<br>43.802<br><0.001	1.327*<br>41.438<br><0.001<br>.560	1.138*<br>26.786<br><0.001<br>.180	1.058*<br>10.963<br><0.001<br>.911	1.058*<br>11.523<br><0.001<br>.460
Colon	36260	Ratio<br>*t*-test<br>MC test<br>Fraction	1.149*<br>44.656<br><0.001<br>.432	1.126*<br>48.604<br><0.001<br>.366	1.177*<br>62.061<br><0.001	1.098*<br>48.784<br><0.001<br>.238	1.093*<br>42.269<br><0.001<br>.872	1.021*<br>9.439<br><0.001<br>.635
Kidney	9825	Ratio<br>*t*-test<br>MC test<br>Fraction	1.071*<br>11.351<br><0.001<br>.326	1.063*<br>12.512<br><0.001<br>.281	1.099*<br>17.191<br><0.001	1.036*<br>9.867<br><0.001<br>.117	1.041*<br>9.186<br><0.001	1.007<br>NSE
Liver	23626	Ratio<br>*t*-test<br>MC test<br>Fraction	1.000<br>NSE	1.021*<br>6.654<br><0.001<br>.238	1.039*<br>10.378<br><0.001	1.009*<br>3.538<br><0.001<br>.098	.995<br>NSE	1.015*<br>5.391<br><0.001
Lung	90050	Ratio<br>*t*-test<br>MC test<br>Fraction	.991<br>NSE	1.005*<br>3.376<br>.081<br>.199	1.039*<br>21.854<br><0.001	.997<br>NSE	1.066*<br>42.629<br><0.001	.973<br>NSE
Ovary	5758	Ratio<br>*t*-test<br>MC test<br>Fraction	.991<br>NSE	.971<br>NSE	.993<br>NSE	.987<br>NSE	.973<br>NSE	1.017<br>2.940
Pancreas	6195	Ratio<br>*t*-test<br>MC test<br>Fraction	1.054*<br>6.687<br><0.001<br>.337	1.038*<br>5.866<br><0.001	1.065*<br>8.702<br><0.001	1.026*<br>5.289<br><0.001<br>.113	.990<br>NSE	1.015<br>NSE

Prostate	5810	Ratio<br>*t*-test<br>MC test<br>Fraction	1.018*<br>2.715<br>.018<br>.143	1.075*<br>14.553<br><0.001<br>.483	1.070*<br>12.186<br><0.001	1.052*<br>12.186<br><0.001<br>.680	1.362*<br>53.228<br><0.001	.941<br>NSE
Rectum	7728	Ratio<br>*t*-test<br>MC test<br>Fraction	1.290*<br>39.488<br><0.001<br>.577	1.241*<br>42.170<br><0.001<br>.509	1.272*<br>42.426<br><0.001<br>.542	1.128*<br>29.901<br><0.001<br>.294	1.022*<br>5.138<br><0.001<br>.043	1.074*<br>16.987<br><0.001<br>.708
Skin	8242	Ratio<br>*t*-test<br>MC test<br>Fraction	1.145*<br>22.752<br><0.001<br>.322	1.142*<br>28.022<br><0.001<br>.239	1.189*<br>35.087<br><0.001<br>.253	1.103*<br>25.986<br><0.001<br>.114	1.252*<br>48.166<br><0.001	.969<br>NSE
Stomach	17810	Ratio<br>*t*-test<br>MC test<br>Fraction	.995<br>NSE	.995<br>NSE	1.056*<br>14.678<br><0.001	1.041*<br>14.868<br><0.001<br>.176	1.116*<br>34.117<br><0.001	.972<br>NSE
Uterus	11903	Ratio<br>*t*-test<br>MC test<br>Fraction	1.160*<br>28.320<br><0.001<br>.473	1.136*<br>30.494<br><0.001<br>.408	1.169*<br>34.360<br><0.001<br>.416	1.106*<br>31.664<br><0.001	1.085*<br>22.785<br><0.001<br>.910	1.059*<br>16.397<br><0.001<br>.687

NSE means that No Significant Excess of mutable motifs was found. The significance of an excess was measured using the Student *t*-test and Monte Carlo (MC) tests. The asterisk denotes that the corresponding $P < 0.0005$ (critical value = 3.291) is a conservative estimate of the critical overall value of the *t*-test taking into account the Bonferroni correction for multiple testing (16 x 6 = 96). "Ratio" is the mean weight of mutated sites divided by the mean weight of non-mutated sites. The predicted fraction of mutations induced by AID/APOBEC proteins ("Fraction") is shown when a significant excess of somatic mutations in mutable motif comparisons was detected; all cases where there was a significant difference between observed and expected distributions ($P > 0.05$) were discarded.

Supplementary Figure S1. AID/APOBEC weight matrices W(b,j).

**Position**	-3 (3)	-2 (4)	-1 (5)	**C** Mutation	+1 (7)	+2 (8)	+3 (9)
**APOBEC1**							
A	-.857	-.314	-2.466	-	.052	.154	.119
T	1.013	.554	1.298	-	.444	.307	.168
G	-1.316	-.820	-4.361	-	-.623	-.381	-.353
C	-.779	.074	-2.571	-	-.196	-.260	-.012
Smax = 5.148							
Smin = -9.409							
**APOBEC3A**							
A	-.160	-.050	-2.615	-	.476	.243	-.039
T	.396	.516	1.268	-	-.090	.256	.219
G	-.559	-1.222	-2.569	-	.098	-.258	-.258
C	.090	.046	-1.917	-	-1.041	-.454	.040
Smax = 4.025							
Smin = -7.082							
**APOBEC3B**							
A	-.032	.261	-2.799	-	.400	.261	-.026
T	.330	.176	1.252	-	.039	.352	.218
G	-.401	-.153	-3.554	-	-.229	-.335	-.273
C	-.030	-.456	-1.294	-	-.429	-.655	.039
Smax = 3.807							
Smin = -7.098							
**APOBEC3C**							
A	.239	-.499	-.332	-	.399	.362	.239
T	.286	1.020	.664	-	.053	.104	.244
G	-.473	-1.390	-.697	-	-.003	.114	-.211
C	-.293	-1.392	-.213	-	-.832	-1.169	-.476
Smax = 3.914							
Smin = -7.233							
**APOBEC3G**							
A	.290	-.933	-3.004	-	.695	.249	.160
T	.488	.043	-1.132	-	-.193	.188	.225
G	-.479	-2.500	-3.982	-	-.254	-.132	-.349
C	-1.071	.954	1.349	-	-1.093	-.481	-.151
Smax = 4.798							
Smin =-10.496							
**AID**							
A	.404	.774	.391	-	.141	.027	.072
T	.012	.218	-.311	-	.068	.095	.031
G	-.321	-2.734	.342	-	.119	.007	-.107
C	-.294	-.944	-.917	-	-.401	-.109	.026
Smax = 2.347							
Smin = -5.001							

Supplementary Figure S2. The overall distribution of fraction of somatic C:G > T:A mutations associated with AID/APOBEC deamination (APOBEC1, APOBEC3A, ABOPEC3B and AID deaminases).

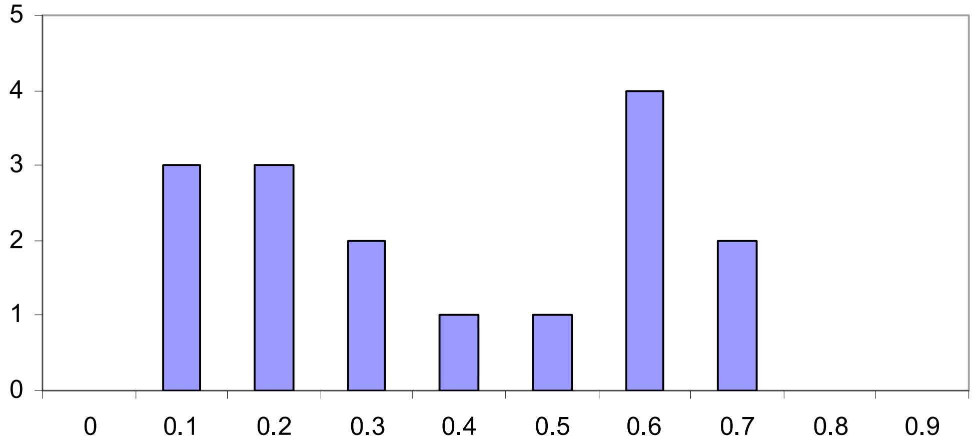

Supplement: Supplementary file 1 [file cancers-11-00211-s001.pdf]
